# Supplementary material for: Effects of β-Phenylethylamine on Psychomotor, Rewarding, and Reinforcing Behaviors and Affective State: The Role of Dopamine D1 Receptors
Source: Int J Mol Sci. 2021 Aug 31;22(17):9485. doi: 10.3390/ijms22179485 (PMC8430604; doi:10.3390/ijms22179485)
Supplement: Supplementary file 1 [file ijms-22-09485-s001.zip › ijms-1355784-supplementary.pdf]

Supplementary Materials

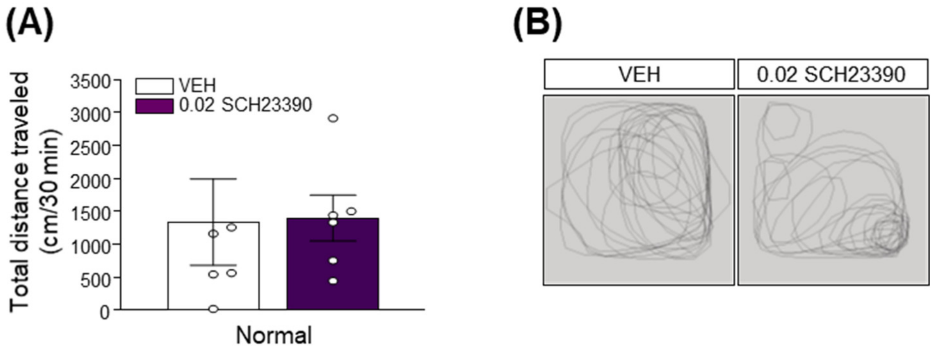

Figure S1. Effect of SCH23390 in normal mice. Total traveled distance for 30 min (A) and representative tracking patterns (black lines in each gray rectangle) for 30 min (B) after acute administration of vehicle or 0.02 mg/kg SCH23390 in normal mice. n = 6 per group.
